# High affinity targeting of CD23 inhibits IgE synthesis in human B cells

**DOI:** 10.1002/iid3.72

**Published:** 2015-07-14

**Authors:** Marc Fellmann, Patrick Buschor, Silvan Röthlisberger, Fabian Zellweger, Monique Vogel

**Affiliations:** ^1^ Department of Immunology, University Clinic RIAUniversity of BernInselspitalSwitzerland

**Keywords:** Allergy, B cells, DARPin, FcϵRII (CD23), IgE

## Abstract

The low‐affinity IgE receptor FcϵRII (CD23) is part of the regulatory system controlling IgE synthesis in human B cells and exists in membrane and soluble forms. Binding of IgE to CD23 has been described to have stabilizing effects and to prevent cleavage of CD23. Previous experiments using anti‐CD23 antibodies reduced IgE synthesis but were difficult to interpret as the antibody Fc part might also mediate feedback mechanisms. The purpose of this study was to investigate the regulatory role of CD23, by using designed ankyrin repeat proteins (DARPins) that specifically recognize CD23. Anti‐CD23 DARPins were isolated by ribosome display and were produced as monovalent and bivalent constructs. Affinities to CD23 were measured by surface plasmon resonance. IgE synthesis and up‐regulation of CD23 in human peripheral B cells were induced by IL‐4 and anti‐CD40 antibody. We assessed CD23 expression and its stabilization by FACS and used an ELISA for detecting soluble CD23. IgE synthesis was measured by ELISA and real‐time PCR. Surface plasmon resonance revealed affinities of the DARPins to CD23 in the pico‐molar range. Anti‐CD23 DARPins strongly inhibited binding of IgE to CD23 and share thus a similar binding epitope as IgE. The DARPins stabilized membrane CD23 and reduced IgE synthesis in an isotype specific manner. Furthermore, the anti‐CD23 DARPins decreased IgE transcript through inhibition of mature Cϵ RNA synthesis suggesting a posttranscriptional control mechanism. This study demonstrates that targeting CD23 alone is sufficient to inhibit IgE synthesis and suggests that a negative signaling occurs directly through the CD23 molecule.

## Introduction

FcϵRII (CD23) is the low affinity receptor for IgE. It is a type II transmembrane glycoprotein consisting of a short cytoplasmic N‐terminus followed by an extracellular part with a stalk region and a *C*‐type lectin‐domain at the C‐terminus, where binding of IgE occurs 1. CD23 appears in a trimeric form due to an α‐helical coiled‐coil structure in the stalk region leading to enhanced binding of IgE (*K*_D_ 3.7 × 10^−^^8^ M) through an avidity effect 2, 3.

In B cells, alternative splicing results in the expression of CD23 in two isoforms (CD23a and CD23b) differing in six or seven amino acids at the N‐terminus 1. CD23a is constitutively expressed in B cells while CD23b, expressed upon stimulation with IL‐4, is present also on monocytes, follicular dendritic cells as well as intestinal epithelial cells 1, 4. Both isoforms were described to have different functions. CD23a on B cells is thought to be associated with endocytosis of IgE‐allergen complexes, whereas CD23b with IgE‐mediated phagocytosis 5, 6. Moreover, CD23 also exists in a soluble form which is derived from the cell‐bound form by cleavage through specific proteases 7. Previous studies have reported that soluble CD23 positively controls IgE synthesis by cross‐linking membrane IgE with CD21 8. It has been hypothesized that binding of IgE or anti‐CD23 antibodies to the lectin domain renders CD23 less susceptible to cleavage by proteases leading to a reduced IgE synthesis. Furthermore, specific inhibition of the protease ADAM 10 has demonstrated a reduction in the synthesis of IgE 8, 9, 10, 11. These data suggest that surface CD23 may be involved in a negative feedback inhibition of IgE synthesis.

The monoclonal chimeric anti‐CD23 antibody, lumiliximab (Biogen Idec, Cambridge, Massachusetts) was shown to reduce IL‐4‐induced IgE synthesis in vitro 12. Furthermore, lumiliximab was demonstrated to reduce serum IgE levels in allergic patients 13 and to mediate apoptosis and anti‐tumor activity in chronic lymphocytic leukemia cells 14. This provides proof of principle that CD23 is a valid target to down‐regulate B cell activation. Previous studies have demonstrated that binding of CD23 by IgE/anti‐IgE immune‐complexes or anti‐CD23 antibodies on a human IgE‐secreting B cell line suppresses IgE synthesis 15. These experiments provide evidence that cross‐linking of CD23 is crucial for the inhibition of IgE synthesis, as no inhibition was observed using monomeric IgE 11, 15. Nevertheless, it remains unknown whether targeting CD23 is sufficient to reduce IgE synthesis as it has been claimed that inhibition of IgE synthesis by anti‐CD23 monoclonal antibodies is Fc‐mediated through cross‐linking of CD23 with the inhibitory FcγRIIb 16.

To investigate the effects of targeting CD23 in more detail we have used non‐immunoglobulin‐like binding scaffolds termed designed ankyrin repeat proteins (DARPin) lacking an Fc‐domain. DARPin binders with high specificities and high affinities have been already isolated against various targets 17, 18. DARPins are expressed in high quantities and in soluble form in *Escherichia coli*; moreover they can be easily fused to each other 19 or to other molecules 20 to generate bispecific binding molecules. Two DARPins were isolated and produced as monovalent and bispecific proteins, both recognizing specifically CD23. These DARPins were able to compete with IgE for binding of CD23 and were tested for their ability to stabilize CD23 and to inhibit IgE synthesis in human B cells.

## Materials and Methods

### Isolation of human B cells

Buffy coats were purchased from the blood donation center (Bern, Switzerland). The study was approved by the local ethics committee. Peripheral blood mononuclear cells (PBMC) were isolated by density gradient centrifugation on Ficoll Paque (GE Healthcare, Chalfont St. Giles, UK). B cells were isolated by negative selection using magnetic beads (StemCell Technologies, Grenoble, France). The average purity of B cells from five independent donors was 94% ± 0.8 (*n* = 5) as determined by flow cytometry (Supplementary Fig. S4A).

### In vitro selection and production of monovalent and bivalent DARPins

The principle of DARPin selection is published elsewhere 21. DARPin libraries were screened by ribosome display to select specific binders against the extracellular part of recombinant CD23 (R&D, Minneapolis, Minnesota) as described previously 19. Briefly, after four selection rounds, binders were produced in *E. coli*. Crude DARPin extracts were screened by ELISA to identify specific binders against CD23 which led to the selection of two specific DARPins. Bivalent and bispecific DARPins were produced using the plasmid pQi‐bi‐2‐2 (kindly provided by Molecular Partners AG, Schlieren, Switzerland). Expression and purification of monovalent, bivalent, and bispecific DARPins was performed as published 19. Additionally, the DARPins were applied to a Superdex 75 column for a size exclusion chromatography (HiLoad 26/60Sudex75, Äkta, Uppsala, Sweden). Plasmid DNA from monovalent, bivalent and bispecific DARPins were sequenced at Microsynth (Balgach, Switzerland).

### Cell culture

B cells were cultured in 48‐well plates at 2 × 10^6^ cells/mL in RPMI 1640 (Gibco, Grand Island, New York) containing 10% FBS (Thermo Scientific, Cramlington, UK), Transferrin 40 µg/mL (Calbiochem, Darmstadt, Germany), mercaptoethanol 50 µM (Sigma–Aldrich, St. Louis, Missouri), Sodium pyruvate 1 mM (Sigma–Aldrich), bovine insulin 4 µg/ml (Sigma–Aldrich), l‐glutamine 2 mM (Gibco), and MEM Non‐essential amino acids 1:100 (Gibco). Cells were activated with IL‐4 20 ng/mL (Peprotech, Rocky Hill, New Jersey) and anti‐CD40 antibody 1 μg/mL (eBioscience, San Diego, California) for up to 13 days. RPMI 8866 cell line was cultured in RPMI 1640 medium (Biochrom, Berlin, Germany) supplemented with 10% FBS. Anti‐CD23 DARPins were added to the cell culture at day 0 at a concentration of 300 nM if not otherwise stated.

### Surface plasmon resonance

Affinities of DARPins were measured on a Biacore X100 instrument (GE Healthcare) with HBS‐EP+ running buffer (GE Healthcare). Recombinant CD23 (Uniprot P06734, amino acids 48‐321) was immobilized on a CM5 biosensor chip using a standard amine coupling kit (GE Healthcare) to an immobilization level of 2400 response units (RU). Binding of anti‐CD23 DARPins was assessed in a twofold serial dilution with at least ten concentrations. Samples were injected for 2 min, followed by 10 min dissociation. Kinetic parameters were calculated with BIAevaluation software (GE Healthcare) using global fitting of the binding data with a 1:1 Langmuir binding.

### Flow cytometry

To visualize CD23, CD20, and CD45 on B cells, cells were stained using Phycoerythrin(PE)‐conjugated mouse anti‐human CD23 IgG1 1:500 (R&D) or with PerCP‐conjugated mouse anti‐human CD20 IgG2b 1:500 (Biolegend, San Diego, California) or with FITC‐conjugated mouse anti‐human CD45 1:500 (Miltenyi Biotec., Bergisch Gladbach, Germany) for 30 min at 4°C, washed and analyzed on guava easyCyte™ Flow cytometer (Merck Millipore, Darmstadt, Germany). In the inhibition assays, the chimeric IgE‐JW8 22 was FITC‐labeled using LYNX Rapid Fluorescein antibody conjugation kit (Bio‐RAD, Kidlington, UK). Cell viability was assessed using annexin V‐APC (Biolegend) and 7‐AAD (Biolegend). Data were analyzed using FLOWJO software (TreeStar Inc., Ashland, Oregon).

### ELISA

Ninety six‐well microtiter plates (Costar, Cambridge, Massachusetts) were coated with monoclonal mouse anti‐human IgE HB235 at 5 µg/mL (ATCC, Manassas, Virgina), polyclonal sheep anti‐human IgG at 5 µg/mL (Binding Site, Birmingham, UK), with sheep anti‐human IgG1 1:1000 (Binding Site), with sheep anti‐human IgG4 1:1000 (Binding Site), with sheep anti‐human IgM 1:1000 (Binding Site), or with monoclonal mouse anti‐human CD23 at 2 µg/mL (R&D) overnight in PBS. Unspecific binding sites were blocked with PBS‐casein (0.15%) for 2 h at 37°C. Cell culture supernatants were incubated for 3 h at 37°C. Detection antibodies were the following: horseradish peroxidase HRP‐conjugated sheep anti‐human IgE 1:1000 (Binding Site), HRP‐conjugated sheep anti‐human IgG 1:2000 (Binding Site), HRP‐conjugated sheep anti‐human IgM 1:1000 (Binding Site) and HRP‐conjugated goat anti‐human CD23 1:500 (R&D). TMB (3,3′,5,5′‐tetramethylbenzidine) was used as substrate and was stopped with 1 M sulphuric acid. Monoclonal human IgE (Sus11) 23, humanized monoclonal IgG omalizumab IgG1 (Xolair®, Novartis, Switzerland) or recombinant human CD23 (Uniprot P06734, amino acids 48‐321) were used for standard curves and concentrations were interpolated (nonlinear regression) using Prism 6.0 software (GraphPad, San Diego, California).

### Real‐time PCR

Total mRNA was isolated using Guanidinum thiocyanate‐phenol‐chloroform extraction (TRI Reagent, MRC, Cincinnati, Ohio). Isolated mRNA was reverse transcribed using random hexamer primers (Applied Biosystems, Branchburg, New Jersey) and Superscript II (Life Technologies, Gaithersburg, Maryland). qPCR was performed using TaqMan Universal Master Mix (Applied Biosystems) on a 7500 Real Time System (Applied Biosystems). Expression was normalized to the endogenous control *β*_2_‐microglobulin and relative expression was determined on 7500 system software (Applied Biosystems). Following primers and probes were used: *β*_2_‐microglobulin, Hs00187842_m1; IgG1, Hs00378340_m1 (Applied Biosystems). IgG4 forward: 5′‐CTGGTCACCGTCTCCTCA‐3′, reverse: 5′‐AGTAGTCCTTGACCAGGCA‐3′, probe: 5′‐FAM‐TTCAAGGGCCCATCGGTCTTCC‐tamra‐3′; IgE forward (mature): 5′‐ACCCTGGTCACCGTCTCC‐3′, forward (germline): 5′‐ACCATCCACAGGCACCAA‐3′, reverse: 5′‐GAGTCACGGAGGTGGCATT‐3′, probe: 5′‐FAM‐CCCTTGACCCGCTGCTGCAAA‐tamra‐3′; CD23a forward: 5′‐TGGAGGAAGGTCAATATTCAGAG‐3′, CD23b forward: 5′‐ATGAATCCTCCAAGCCAGGA‐3′, reverse: 5′‐GCCACAGGAGAAGCAGAGTC‐3′, probe: 5′‐FAM‐GGTGTTGCAGGCGTGGGACT‐tamra‐3′ were ordered at Microsynth (Balgach, Switzerland).

## Results

### Selection of DARPins specific for CD23

Binders recognizing recombinant CD23 were isolated from two DARPin libraries coding for either two (N2C) or three (N3C) randomized ankyrin repeat modules. Binders were expressed and crude DARPin extracts were screened for specificity in an ELISA. Two N3C DARPins termed D86 and D89, displaying no reactivity to control proteins, were chosen for further investigation and expressed in *E. coli* as monovalent (D86, D89) and bivalent (D86‐86, D89‐89) proteins.

In order to investigate whether the two DARPin molecules D86 and D89 recognize different epitopes on CD23, we performed an assay on surface plasmon resonance. The chip surface was coated with bivalent DARPin D86‐86 (Supplementary Fig. S1) and subsequently incubated with recombinant CD23 until the sensogram reached a stable response. DARPin D89 was then injected and was still able to bind to CD23 indicating that the two DARPins, D86 and D89, recognize different epitopes on CD23 which is a prerequisite to generate bispecific DARPins. Monovalent DARPins D89 and D86 were then fused and expressed as a bispecific DARPin (D89‐86). SDS‐PAGE showed that DARPins D86, D86‐86 and D89‐86 run as monomeric molecules, while DARPins D89 and D89‐89 tend to form dimers (Supplementary Fig. S2).

### Determination of kinetic parameters of anti‐CD23 DARPins

Kinetic parameters of anti‐CD23 DARPins were assessed by surface plasmon resonance on immobilized CD23 (Supplementary Fig. S3 and Table 1). Monovalent DARPins showed similar association and dissociation rates, resulting in low micromolar equilibrium dissociation constants (Table 1). Compared to monovalent DARPins, bivalent D86‐86 and bispecific D89‐86 DARPins had an almost unaltered on‐rate but a lower off‐rate resulting in increased *K*_D_ values of nearly four orders of magnitude. These improved affinities might be mainly due to increased avidity. Interestingly, bivalent DARPin D89‐89 has a faster off‐rate, which results in an affinity ten times less compared with the other bivalent and bispecific molecules.

**Table 1:** Binding affinities of anti‐CD23 DARPins for human CD23

Name	Structure	*k*_a _ [M^−1 ^s^−^^1^]	*K*_d_ [s^−^^1^]	*K*_D_ [M]
D86	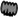	1.28 × 10^7^	3.69 × 10^−^^1^	2.88 × 10^−^^8^
D89	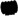	2.95 × 10^7^	4.53 × 10^−^^1^	1.54 × 10^−^^8^
D86‐86	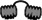	5.79 × 10^7^	1.54 × 10^−^^4^	2.66 × 10^−^^12^
D89‐89	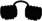	6.99 × 10^8^	8.94 × 10^−^^3^	1.28 × 10^−^^11^
D89‐86	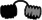	2.79 × 10^7^	4.46 × 10^−5^	1.60 × 10^−^^12^

### Inhibition of IgE binding to membrane CD23 by anti‐CD23 DARPins

**Figure 1:**
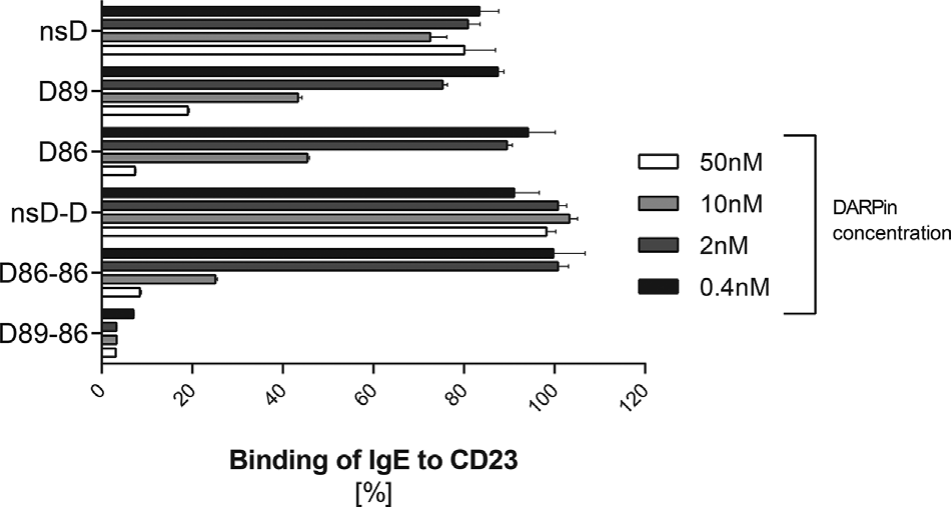
Flow cytometry analysis showing the inhibition of IgE binding to CD23 by anti‐CD23 DARPins. Anti‐CD23 monovalent (D89, D86), bivalent (D86‐86), and bispecific (D89‐86) DARPins at different concentrations were mixed with FITC‐labeled IgE at a saturating concentration of 25 nM and were added to RPMI 8866 cells for 30 min at 4°C. Two unspecific monovalent (nsD) and bivalent (nsD‐D) DARPins were used as controls. The results are expressed in percentage of IgE binding to CD23.

To assess the capacity of the anti‐CD23 DARPins to block binding of IgE to CD23, an inhibition assay was performed using the lymphoblastoid B cell line RPMI 8866. From the affinity experiment, there was evidence that the bispecific and bivalent DARPins (D89‐86, D86‐86) had the highest affinities. Therefore, the experiment was performed by mixing monovalent (D86, D89), bivalent (D86‐86), and bispecific (D89‐86) DARPins at different concentrations with a saturating concentration (25 nM) of FITC‐labeled IgE before adding to the cells. As shown in Figure 1, both monovalent DARPins D86 and D89 inhibited the interaction of IgE with CD23 to more than 80% only at the highest concentration. Compared to the monovalent DARPins, bispecific DARPin D89‐86 showed an inhibition of more than 80% already at the lowest concentration (0.4 nM). Surprisingly, the inhibitory capacity of bivalent DARPin D86‐86 was not better than that of the monovalent DARPins. This might indicate a steric hindrance of the bivalent DARPin in binding to membrane CD23. The binding of IgE to CD23 was not inhibited by non‐specific bivalent (nsD‐D) and monovalent (nsD) control DARPins (Fig. 1). These results showed that the bispecific DARPin (D89‐86) performed as the most potent inhibitor and was chosen for subsequent in vitro cell assays.

### Anti‐CD23 DARPins stabilize membrane CD23

**Figure 2:**
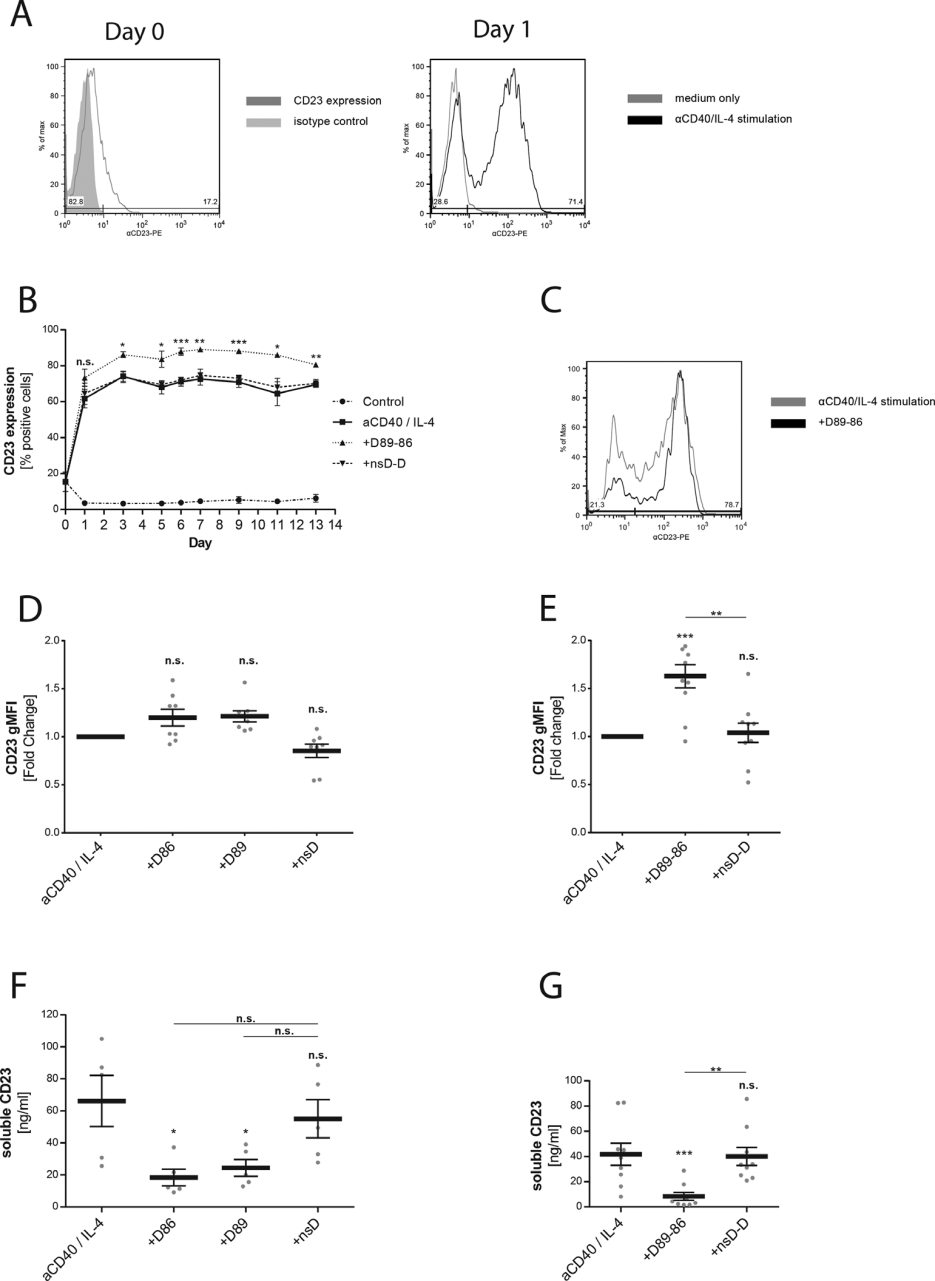
Anti‐CD23 DARPins stabilize membrane CD23 on human B cells. (A) Surface CD23 of purified human B cells was stained before (Day 0) and after overnight (Day 1) incubation with and without IL‐4 and anti‐CD40 antibody. (B) Time course of surface CD23 expression in human B cells cultured in medium or IL‐4 and anti‐CD40 with or without the bispecific anti‐CD23 DARPin (D89‐86) or the bispecific control DARPin (nsD‐D) added at day 0. The cells were stained with PE‐labelled anti‐CD23 antibody and analysed by flow cytometry. The results are expressed as percent CD23 positive cells. (C) Representative histogram from one donor after stimulation with D89‐86 in comparison with IL‐4/anti‐CD40 stimulation alone. The cells were stained at day 13 with PE‐labeled anti‐CD23 antibody. (D, E) Surface level of CD23 on purified human B cells cultured in the presence the anti‐CD23 DARPins or with the non‐specific control DARPins (nsD and nsD‐D) assessed at day 13. The results are expressed as normalized geometric mean fluorescence intensity (gMFI) relative to cells treated with IL‐4 and anti‐CD40 antibody alone. (F, G) Purified human B cells were cultivated with IL‐4 and anti‐CD40 alone or in the presence of the anti‐CD23 DARPins D86, D89 or D89‐86 added at day 0. No effects were observed using the non‐specific control DARPins (nsD and nsD‐D). Cell supernatants were collected at day 13 and soluble CD23 levels were measured by ELISA. Each point represents an individual donor. Shown are means ± SEM, statistical significance was calculated using 1‐way ANOVA with Dunnet's post‐comparison test and considered significant if *P* < 0.05.

As targeting CD23 by IgE has been shown to stabilize surface CD23 24, 25, we investigated whether the bispecific DARPin D89‐86 displays the same effect on primary human B cells isolated from peripheral blood. B cells were stimulated with IL‐4 plus anti‐CD40 antibody in order to induce class‐switch toward IgE synthesis as well as to up‐regulate expression of CD23. Initial expression of CD23 was detected in 10–20% of isolated B cells at day 0 and was increased to 70–80% already after 24 h of stimulation where it remained constant for the following 13 days (Fig. 2A and B). In the presence of DARPin D89‐86, the amount of CD23 positive cells was significantly enhanced during the time course of the experiment compared to control samples (Fig. 2B and C). Furthermore, when added at different concentrations at the onset of the experiment, the effect of D89‐86 on stabilization was found to be dose dependent (Supplementary Fig. S5E). To obtain more evidence for the effect of DARPin D89‐86 on the expression of surface CD23, we purified B cells from at least five donors and compared the effect of D89‐86 with its monovalent analogs after 6 and after 13 days of culture. The expression of CD23 was normalized to IL‐4/anti‐CD40‐stimulated samples. No difference in the expression of CD23 was observed with monovalent DARPins D86 and D89 at day 6 (Supplementary Fig. S5A) and at day 13 (Fig. 2D). In contrast, with the bispecific DARPin D89‐86, a significant increase in CD23 expression was found after 6 (Supplementary Fig. S5B) and 13 days (Fig. 2E).

As expected, supernatants of IL‐4/anti‐CD40‐stimulated B cells showed an increase of soluble CD23 in comparison with unstimulated samples. Monovalent DARPins D89 and D86 as well as bispecific DARPin D89‐86 significantly decreased the amount of soluble CD23 after 6 (Supplementary Fig. S5C and D) and 13 (Fig. 2F and G) days of culture. However, the reduction was more pronounced with bispecific DARPin D89‐86. For comparison, the control DARPins (nsD and nsD‐D) did neither influence the expression of membrane CD23, nor the amount of soluble CD23 released into the supernatants. Altogether, these findings indicate that anti‐CD23 DARPins stabilize surface CD23 and confirm the relationship between stabilization and the release of soluble CD23.

### Influence of anti‐CD23 DARPins on CD23 mRNA expression

**Figure 3:**
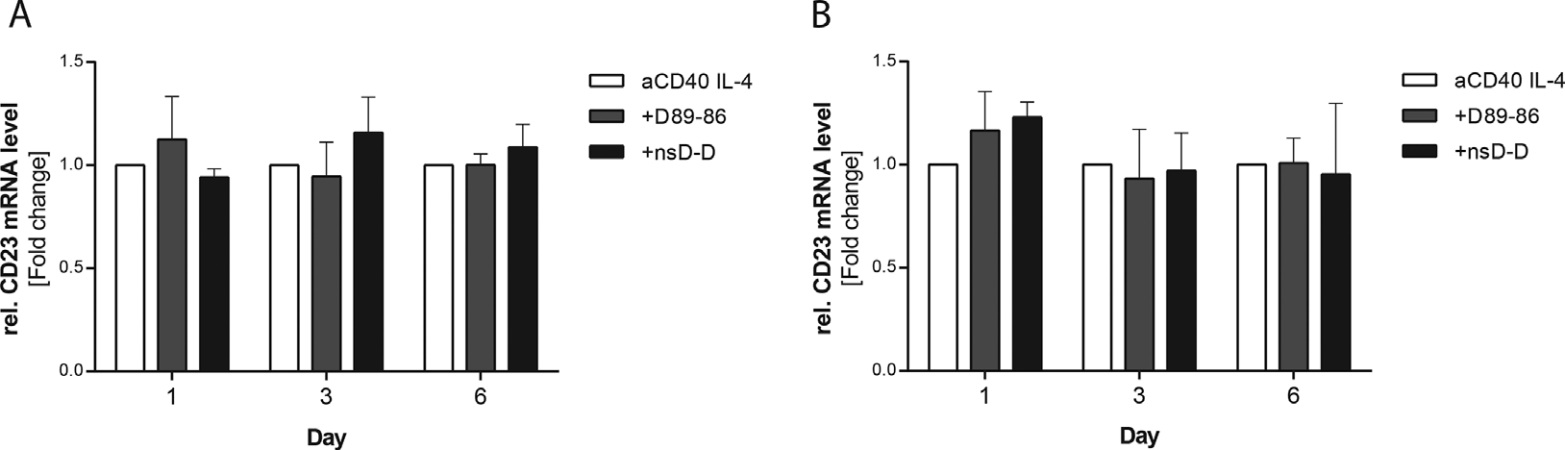
Effect of the bispecific anti‐CD23 DARPin D89‐86) on CD23 mRNA transcription. Human B cells were cultured with IL‐4 and anti‐CD40 alone or in the presence of the bispecific anti‐CD23 DARPin or bispecific control DARPin (nsD‐D) added at day 0. Expression of CD23a (A) and CD23b (B) mRNA was quantified by real time RT‐PCR after 1, 3, and 6 days. Relative expression is shown as fold change compared to anti‐CD40/IL‐4‐stimulated samples (ΔΔCt). Bars represent means ± SEM of at least three different donors. No significant differences were observed by using 1‐way ANOVA with Dunnet's post‐comparison test.

Next, we investigated whether the stabilized CD23 expression induced by DARPin D89‐86 in IL‐4/anti‐CD40‐stimulated B cells influences CD23 mRNA. Although B cells show a constitutive expression of isoform a, both isoforms are up‐regulated by IL‐4 (data not shown). Incubation of B cells with bispecific DARPin D89‐86 did not significantly influence expression of CD23 mRNA, indicating a post‐transcriptional mechanism of membrane CD23 stabilization (Fig. 3A and B).

### Anti‐CD23 DARPins reduce IgE synthesis in human B cells in an isotype‐specific manner

**Figure 4:**
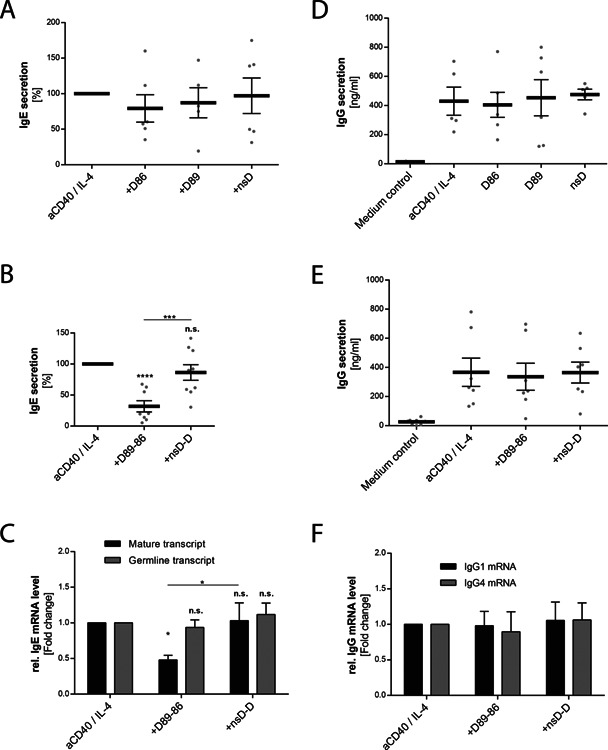
Anti‐CD23 DARPins reduce IgE synthesis in an isotype‐specific manner. IL‐4 and anti‐CD40 stimulated human B cells were cultivated with the anti‐CD23 DARPins D86, D89, or D89‐86 or with the non‐specific control DARPins (nsD and nsD‐D) added at day 0. Cell supernatants were collected at day 13 and IgE (A,B) and total IgG (D,E) levels were measured by ELISA. Because of high donor variability, the results for IgE were normalized to cultures stimulated with IL‐4 and anti‐CD40 alone. The units represent the percentage of IgE secretion compared to the cultures stimulated with IL‐4 and anti‐CD40 antibody alone (G) Germline and mature IgE mRNA transcripts were analyzed after 6 days of culture by real time RT‐PCR. (F) IgG1 (*n* = 4) and IgG4 (*n* = 5) mRNA expression in naïve B cells was analyzed after 6 days of culture by real‐time PCR (ΔΔCt). Relative expression is shown as fold change compared to anti‐CD40/IL‐4‐stimulated samples (ΔΔCt). Shown are means ± SEM, statistical significance was calculated using 1‐way ANOVA with Dunnet's post‐comparison test and considered significant if *P* < 0.05.

Having demonstrated stabilization of membrane CD23 by targeting CD23, we investigated the effect of anti‐CD23 DARPins on IgE synthesis in B cells after 13 days of IL‐4/anti‐CD40 stimulation. A significant inhibition of IgE synthesis was observed when B cells were incubated with bispecific DARPin D89‐86 (Fig. 4B). In contrast, only a minimal reduction of IgE synthesis was observed with monovalent DARPins D86 and D89 that did not reach statistical significance (Fig. 4A). To investigate whether bispecific DARPin D89‐86 influences the level of transcription, we examined the germline Cϵ mRNA transcript (ϵGLT) and mature mRNA production by real‐time qPCR at day 6. Figure 4C shows that the expression of the mature transcript was significantly reduced with bispecific DARPin D89‐86, thereby confirming the observed reduction of IgE synthesis. However, ϵGLT was not reduced after 6 days (Fig. 4C). As ϵGLT can be detected earlier, we analyzed its expression after 3 days but DARPin D89‐86 showed no inhibitory effect on ϵGLT (data not shown).

In order to assess the effect of DARPins on other Ig isotypes, we measured total IgG, IgG1, IgG4, and IgM synthesis in IL‐4/anti‐CD40‐stimulated B cells after 13 days. No significant reduction in the synthesis of these isotypes was observed with either monovalent D86 or D89 (Fig. 4D) or with bispecific D89‐86 (Fig. 4E and Supplementary Fig. S6A–C) DARPins. We further assessed IgG1 and IgG4 mRNA transcripts at day 6 to investigate potential effects of D89‐86 at the level of mRNA transcription. As peripheral B cells might contain already switched B cells producing IgG, human peripheral naïve B cells were isolated and stimulated with IL‐4 plus anti‐CD40. Indeed, no reduction of either IgG1 or IgG4 mRNA expression was observed upon stimulation with bispecific DARPin D89‐86 (Fig. 4F) indicating that the effect of the bispecific DARPin on IgE synthesis was isotype‐specific.

### Anti‐CD23 DARPins do not induce cell death in human B cells

**Figure 5:**
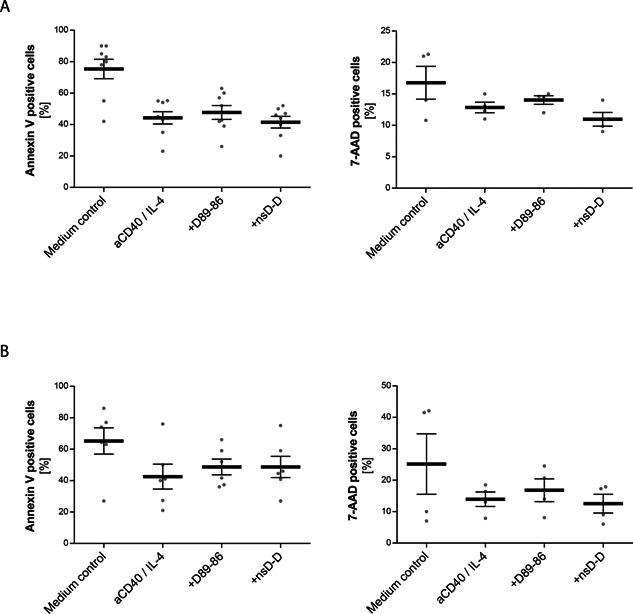
Anti‐CD23 DARPins did not induce apoptosis in human B cells. Human B cells were cultivated with IL‐4 and anti‐CD40 alone or in the presence of the bispecific anti‐CD23 DARPin (D89‐86) or the bispecific control DARPin (nsD‐D) added at day 0. Cells were stained with 7‐AAD and FITC‐conjugated Annexin V after 6 (A) and 13 days (B) and analyzed by flow cytometry. Results are expressed as percent positive cells and are means ± SEM of at least three donors.

It has been previously demonstrated that the anti‐CD23 antibody, lumiliximab, can induce apoptosis in chronic lymphocytic leukemic cells 14. Therefore, we hypothesized that inhibition of IgE synthesis could be due to the induction of apoptosis. To determine whether DARPin D89‐86 induces apoptosis, B cells were incubated with IL‐4 plus anti‐CD40 in the presence and absence of DARPin D86‐89 and the control DARPin (nsD‐D). After 6 days (Fig. 5A) and after 13 days (Fig. 5B) of culture, B cells were stained with the apoptosis marker annexin V (Left panel) or with the cell death marker 7‐AAD (Right panel). Flow cytometry demonstrated that the DARPin D89‐86 did not significantly enhance the percentage of apoptotic cells compared to cells treated with IL‐4 plus anti‐CD40 alone or in the presence of the control DARPin. As apoptosis can be detected at earlier time points, cell viability was investigated after 3 days but no significant reduction was observed (data not shown).

## Discussion

Previous studies have addressed the effect of targeting CD23 using anti‐CD23 antibodies or IgE‐immune complexes 11, 15; however the importance of targeting CD23 per se has not been properly evaluated. In this study, we used the DARPin technology to select binders that recognize CD23 with exclusive specificity and with high affinity. Two DARPins were selected that recognized two different epitopes on CD23 and were able to interfere with IgE binding on CD23. It has been shown that the binding site of IgE on CD23 lies in the lectin “head” domain 3. Therefore, we speculated that the epitopes recognized by DARPins D89 and D86 are clearly distant from each other but overlapped that of IgE. Genetically linking and producing the DARPins D89 and D86 in a bispecific format resulted in a molecule with an enhanced affinity in the picomolar range and a stronger capacity to inhibit binding of IgE to membrane CD23.

Our result showed that the bispecific DARPin D89‐86 significantly enhanced CD23 surface expression and reduced the amount of soluble CD23 released into the cell supernatants. A similar effect has been observed with the natural ligand, IgE, that increases cell surface expression by preventing the release of soluble CD23 26. In contrast, monoclonal anti‐CD23 antibodies that bind to the lectin head domain have been shown to decrease surface expression of CD23 by inducing endocytosis 5. However, like IgE and anti‐CD23 antibodies, D89‐86 did not alter expression of CD23 mRNA levels of either CD23a or CD23b isoform. A likely explanation is that bispecific DARPin D89‐86 induces a conformational change in CD23 that promotes oligomerization of CD23, rendering it less susceptible to proteolysis. This has already been observed by Gould et al. who suggested an allosteric mechanism by which an anti‐CD23 antibody recognizing the lectin domain, or IgE, stabilizes membrane CD23 whereas anti‐CD23 antibodies which recognize the stalk region exert the opposite effect 24, 27.

As mentioned, it has already been postulated that binding CD23 on IgE secreting cells by IgE/anti‐IgE immune‐complexes directly supresses IgE synthesis 15. However, in that system it cannot be excluded that binding of the anti‐IgE antibody to membrane IgE mediates the IgE suppression. Furthermore, studies using the chimeric anti‐CD23 antibody, lumiliximab, have claimed that the reduced IgE synthesis requires co‐aggregation with the inhibitory Fcγ‐receptor 16. Furthermore, it has been reported that F(ab)_2_ of murine monoclonal anti‐CD23 antibodies showed no or only marginal effects on in vitro IL‐4‐induced IgE synthesis in peripheral blood lymphocytes 24. In contrast to those studies, we showed that targeting CD23 with D89‐86 significantly reduced IgE synthesis. This can be explained by the fact that upon epitope specificity targeting CD23 may act differently on IgE production as it has been described previously with monoclonal antibodies recognizing distinct epitopes on CD23 28. Furthermore, in contrast to F(ab)_2_ molecules that recognize with both arms the same epitope, our bispecific DARPin recognizes two epitopes leading to a more efficient stabilization of CD23 on the cell surface. This hypothesis is sustained by the fact that only the bispecific DARPin D89‐86, that efficiently stabilizes CD23 compared to the monovalent anti‐CD23 DARPins, reduced IgE synthesis. This result is in accordance with the observation that protease inhibitors that block cleavage of CD23 down‐regulate IgE synthesis in vitro 8.

It was still unclear whether stabilization of CD23 on B cells is associated with a reduced epsilon class switch recombination. Earlier work has shown that a monoclonal anti‐CD23 antibody may have to cross‐link with the FcγRIIb to block germline Cϵ transcription 16. Our results showed decreased levels of the mature IgE transcript, whereas epsilon germline transcript (ϵGLT) was not affected. It is likely that binding of bispecific DARPin to CD23 does not affect class switch at the stage of germline Cϵ transcription but rather inhibits IgE synthesis after class‐switch recombination (CSR) has occurred. Neither inhibition of IgG synthesis nor decreased IgG1 and IgG4 mRNA levels upon stimulation with bispecific DARPin were observed suggesting an isotype‐specific control for IgE synthesis through a postswitch mechanism.

Additional experiments showed that targeting CD23 with bispecific DARPin does not induce cell apoptosis but rather induces a state of anergy as it has been postulated for the humanized anti‐human IgE antibody, omalizumab 29. Induction of apoptosis in vitro might require extensive cross‐linking as it has been shown for the chimeric anti‐CD23 antibody, lumiliximab, by using a secondary antibody. In vivo, such cross‐linking most probably occurs through binding of the Fc‐domain to Fcγ‐receptors on NK cells or other effector cells 14. Whether extensive cross‐linking by increasing the valency of bispecific DARPin will lead to apoptosis remains to be investigated.

Considering the suppressive effect of the bispecific DARPin on IgE synthesis, we propose three possible mechanisms. First, through its high affinity the bispecific DARPin stabilizes CD23 and avoids the release of soluble CD23 thereby preventing the positive effect of soluble CD23 on IgE synthesis. This view is supported by the fact that bispecific DARPin enhances the surface expression of CD23 whereas monovalent DARPins, having lower affinities, were less effective in reducing cleavage of CD23. Second, bispecific DARPin D89‐86 might deliver a negative signal through cross‐linking surface CD23 as it has been proposed with IgE‐immune‐complexes 15. At the cell surface, CD23 is trimeric and analysis of the crystal structure of IgE bound to CD23 has revealed a topology that does not allow IgE to engage two lectin “head” domains within one CD23 molecule but might cross‐link two membrane CD23 molecules 30. Bispecific DARPin D89‐86 contains a flexible [Gly_4_‐Ser]_4_ linker of approximately 7 nm whose length does most likely not allow intramolecular cross‐linking of the CD23 “head” domains, but could cross‐link two membrane CD23 molecules. Although ITIM signaling through CD23 is still not known, this may lead to a negative signal through CD23. Third, the bispecific DARPin inhibits IgE synthesis by binding to and neutralizing soluble CD23. The importance of soluble CD23 was reported by Gould et al. demonstrating that soluble CD23 positively influences IgE synthesis by cross‐linking membrane IgE and CD21 8. Against this view is that monovalent DARPins, which can bind to soluble CD23, have no inhibitory effect. This further supports the hypothesis that bispecific DARPins are not acting by preventing the binding of soluble CD23 but by binding to membrane CD23.

In conclusion, we have demonstrated that targeting CD23 with a high affinity bispecific DARPin is sufficient to inhibit IgE production and does not require engagement of an inhibitory Fcγ‐receptor. This effect was mediated by a post switch mechanism that does not affect ϵGLT. Furthermore, our data demonstrated the importance of high affinity binding to CD23 for stabilization and revealed a remarkable relationship with down‐regulation of IgE synthesis. Additionally, we speculate that bispecific DARPin has some capacity for signaling but further studies are required to elucidate signal transduction pathways.

## Conflicts of Interest

The University Institute of Immunology at the University of Bern has a research collaboration agreement with Molecular Partners AG (MPAG), who own the intellectual property on the DARPin technology used in this study. All the authors declare no competing financial interests.

## Supporting information

Additional supporting information may be found in the online version of this article at the publisher's web‐site.

Supporting Data

**Figure S1.** Epitope specificities of anti‐CD23 DARPins. The chip was saturated with bivalent DARPin D86‐86, which has a low off‐rate dissociation constant.
**Figure S2.** Protein analysis of anti‐CD23 DARPins. Anti‐CD23 DARPins (1.5 μg) were visualized on a 15% SDS‐PAGE stained with Coomassie.
**Figure S3.** SPR sensorgrams for the assessment of binding kinetics of anti‐CD23 DARPins to CD23.
**Figure S4.** Expression of CD20 and CD23 on freshly isolated B cells.
**Figure S5.** Effects of anti‐CD23 DARPins on surface CD23 after 6 days of culture.
**Figure S6.** Effects of anti‐CD23 DARPins on different immunoglobulin isotypes.
