# Supplementary material for: High affinity targeting of CD23 inhibits IgE synthesis in human B cells
Source: Immun Inflamm Dis. 2015 Jul 14;3(4):339–49. doi: 10.1002/iid3.72 (PMC4693728; doi:10.1002/iid3.72)
Supplement: Supplementary file 1 — Supporting Data [file IID3-3-339-s001.docx]

**Supporting information**

**Methods**

*SDS-PAGE Analysis*

Anti-CD23 DARPins (1.5 μg) were separated by 15% SDS-PAGE for testing their potential to form aggregates. PageRuler prestained protein ladder was used as marker (Thermo Scientific, Cramlington, UK). Gel was stained for 10 minutes using Coomassie Brilliant Blue.

*Surface Plasmon Resonance (SPR) for epitope mapping*

Binding epitopes of DARPins D86 and D89 were assessed on surface plasmon resonance (Biacore X100, GE Healthcare). DARPin D86-86 was immobilized on a CM5 biosensor chip using a standard amine coupling kit (GE Healthcare) to an immobilization level of 2000 response units (RU). Recombinant CD23 at 1000 nM (Uniprot P06734, extracellular domain, amino acids 48-321) was injected for 2 minutes and washed until a stable binding of approximately 100 RU was achieved. Subsequently, DARPins D89 or D86 (1000 nM) were injected for 1 min.

**Figure legends**

**Figure S1**. Epitope specificities of anti-CD23 DARPins. The chip was saturated with bivalent DARPin D86-86, which has a low off-rate dissociation constant. CD23 (1000 nM) was then injected for 2 min until a stable binding was obtained. DARPin D89 (1000 nM) or DARPin D86 (1000 nM) were subsequently injected for 1 min. DARPin D86 showed no additional binding to CD23.

**Figure S2**. Protein analysis of anti-CD23 DARPins. Anti-CD23 DARPins (1.5 ug) were visualized on a 15% SDS-PAGE stained with Coomassie. PageRuler prestained protein ladder was used as marker.

**Figure S3**. SPR sensorgrams for the assessment of binding kinetics of anti-CD23 DARPins to CD23. Recombinant CD23 was immobilized to a response unit of 2400RU. Binding of anti-CD23 DARPins was analyzed using 2-fold serial dilutions. DARPins were injected for 2 min, followed by 10 min dissociation. Kinetic parameters were calculated with BIAevaluation software (Table 1) using global fitting of the binding data with a 1:1 Langmuir binding.

**Figure S4**. Expression of CD20 and CD23 on freshly isolated B cells. (A) B cells were stained for CD45 and for CD20 in order to assess puritiy after isolation and analyzed by FACS as shown as dot plot from one representative donor (left panel) and as histogram compared to unstained control (right panel). (B) CD23 expression in isolated B cells was analyzed by FACS and shown as dot plot in combination with staining for CD45 (left panel) as well as with CD20 (right panel).

**Figure S5**. Effects of anti-CD23 DARPins on surface CD23 after 6 days of culture.

(A, B) Isolated human B cells were cultured with IL-4 and anti-CD40 antibody. Surface expression of CD23 was assessed after 6 days of culture with the anti-CD23 DARPins (300 nM) D86, D89, D89-86 or with the non-specific control DARPins (nsD and nsD-D) added at day 0. The results are expressed as normalized geometric mean fluorescence intensity (gMFI) relative to cells treated with IL-4 and anti-CD40 antibody alone. (C, D) Cell supernatants of B cells cultured with IL-4 and anti-CD40 antibody in the presence of the anti-CD23 DARPins D86, D89 and D89-86 were analyzed for soluble CD23 by ELISA. For comparison, non-specific DARPins (nsD and nsD-D) were used as control. (E) Dose-dependent stabilization of CD23 by DARPin D89-86 after 6 days of culture with IL-4/anti-CD40-stimulated B cells. DARPins were added at day 0 and surface CD23 expression was measured by FACS. Shown are means ± SEM of at least 3 individual donors, statistical significance was calculated using 1-way ANOVA with Dunnet’s post-comparison test and considered significant if p < 0.05.

**Figure S6**. Effects of anti-CD23 DARPins on different immunoglobulin isotypes. (A – C) IL-4/anti-CD40-stimulated B cells were cultured for 13 days with or without the anti-CD23 DARPin D89-86 or the non-specific DARPin (nsD-D). Cell supernatants were tested for IgM, IgG1 or IgG4 by ELISA. Shown are means ± SEM from at least 3 individual donors. No statistical significant difference was observed between samples cultured with the anti-CD23 DARPin D89-86 or samples stimulated with IL-4/anti-CD40 only. (D) Cell supernatants of IL-4/anti-CD40-stimulated B cells cultured with or without the anti-CD23 DARPin D89-86 or the non-specific DARPin (nsD-D) after 13 days were tested for IgE by ELISA. Shown are means ± SEM in ng/ml of at least 3 individual donors, statistical significance was calculated using 1-way ANOVA with Dunnet’s post-comparison test and considered significant if p < 0.05.
