# Supplementary figures and images for: High affinity targeting of CD23 inhibits IgE synthesis in human B cells
Source: Immun Inflamm Dis. 2015 Jul 14;3(4):339–49. doi: 10.1002/iid3.72 (PMC4693728; doi:10.1002/iid3.72)

Figure S1

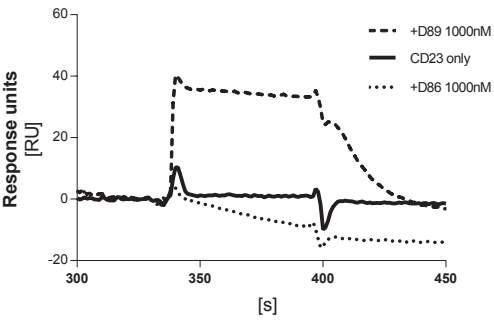

Figure S2

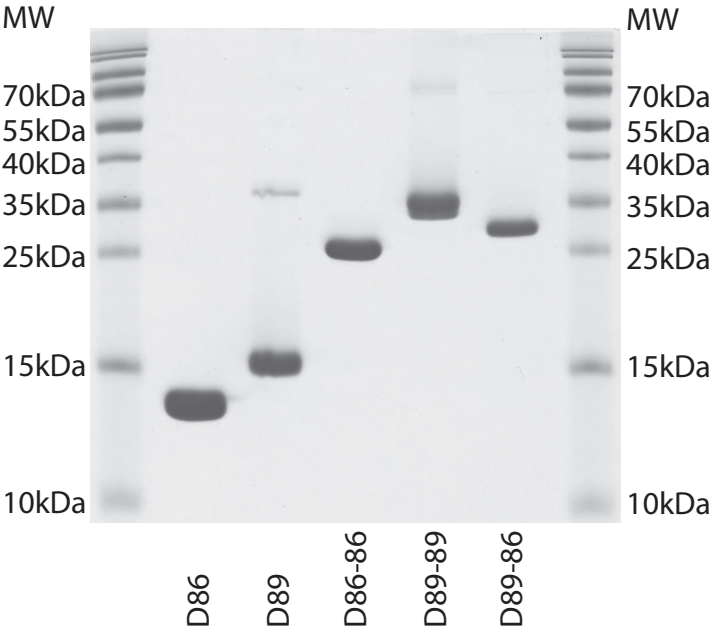

Figure S3

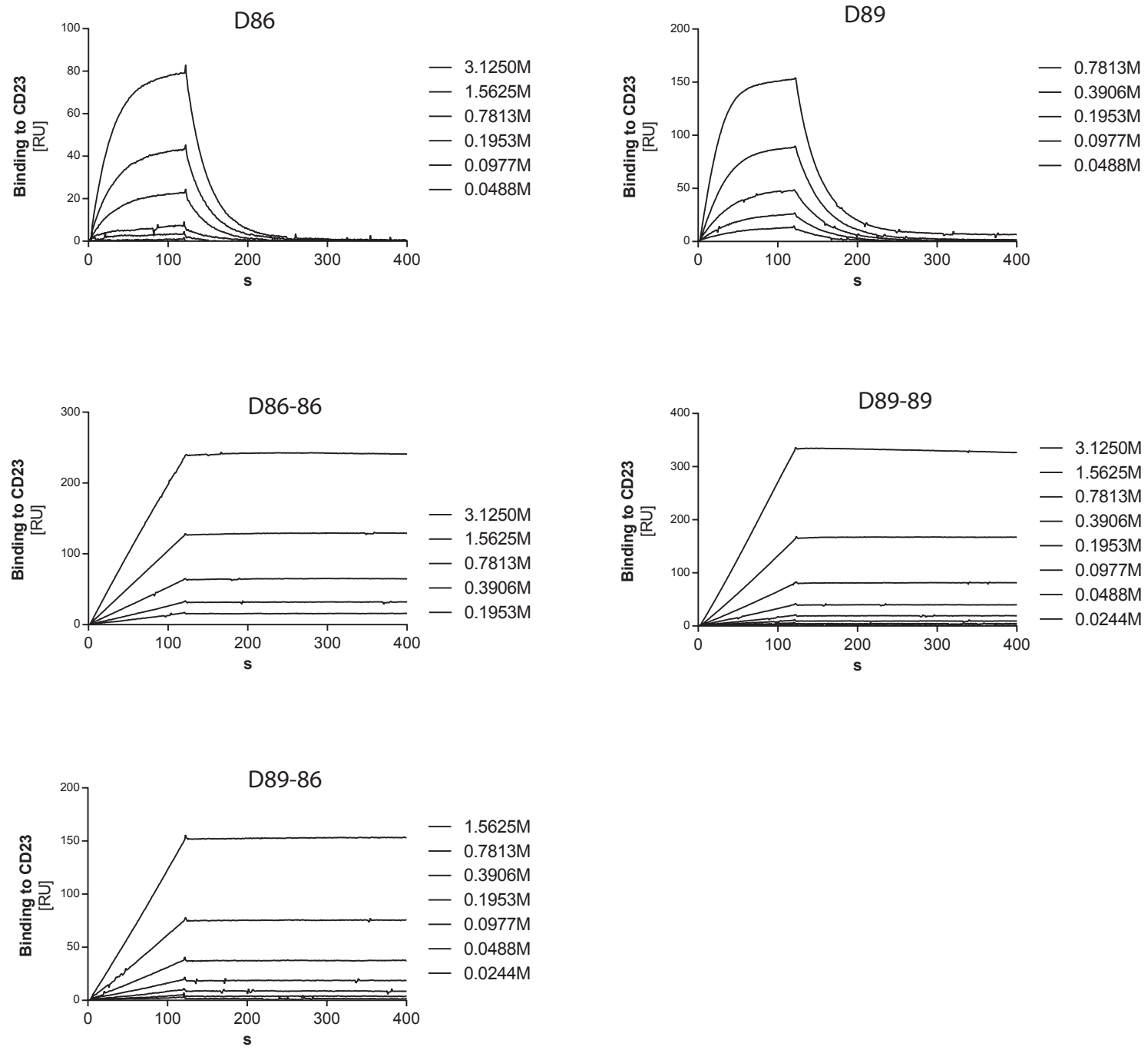

Figure S4

A

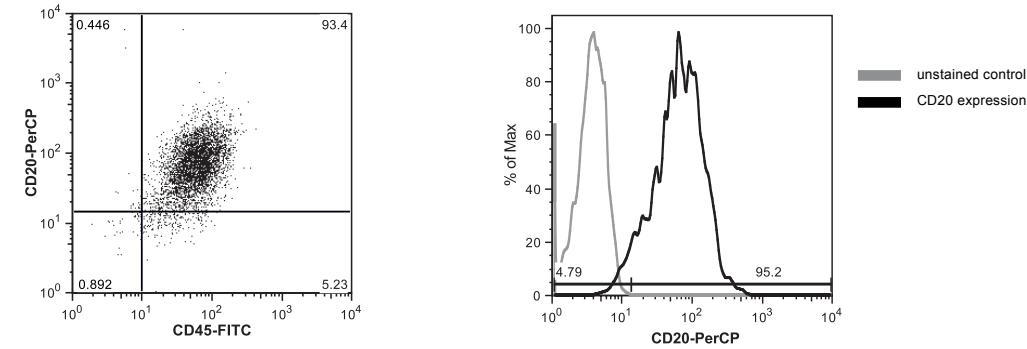

B

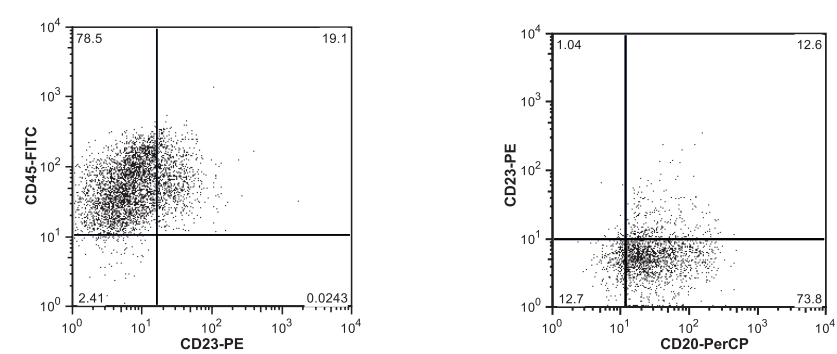

Figure S5

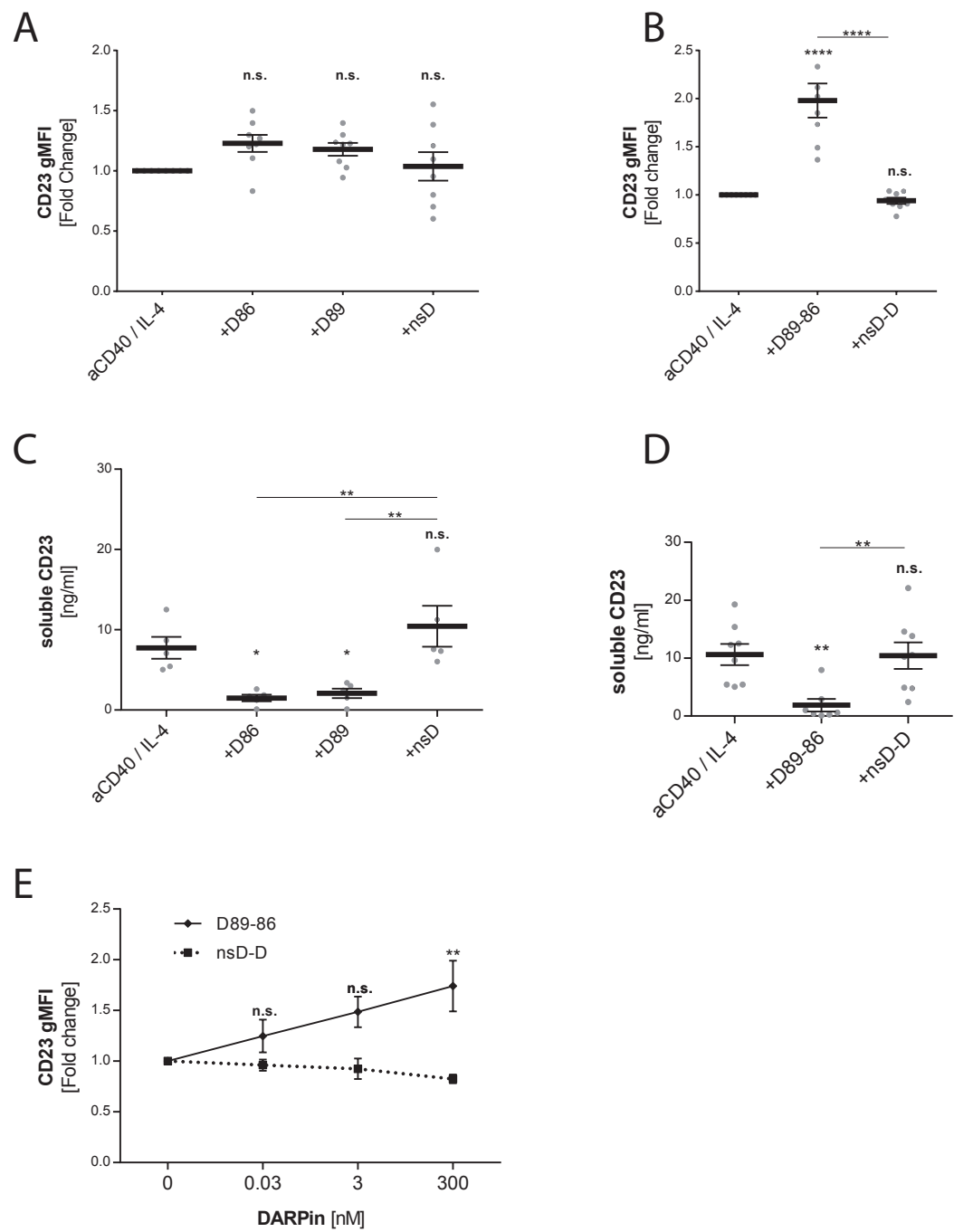

Figure S6

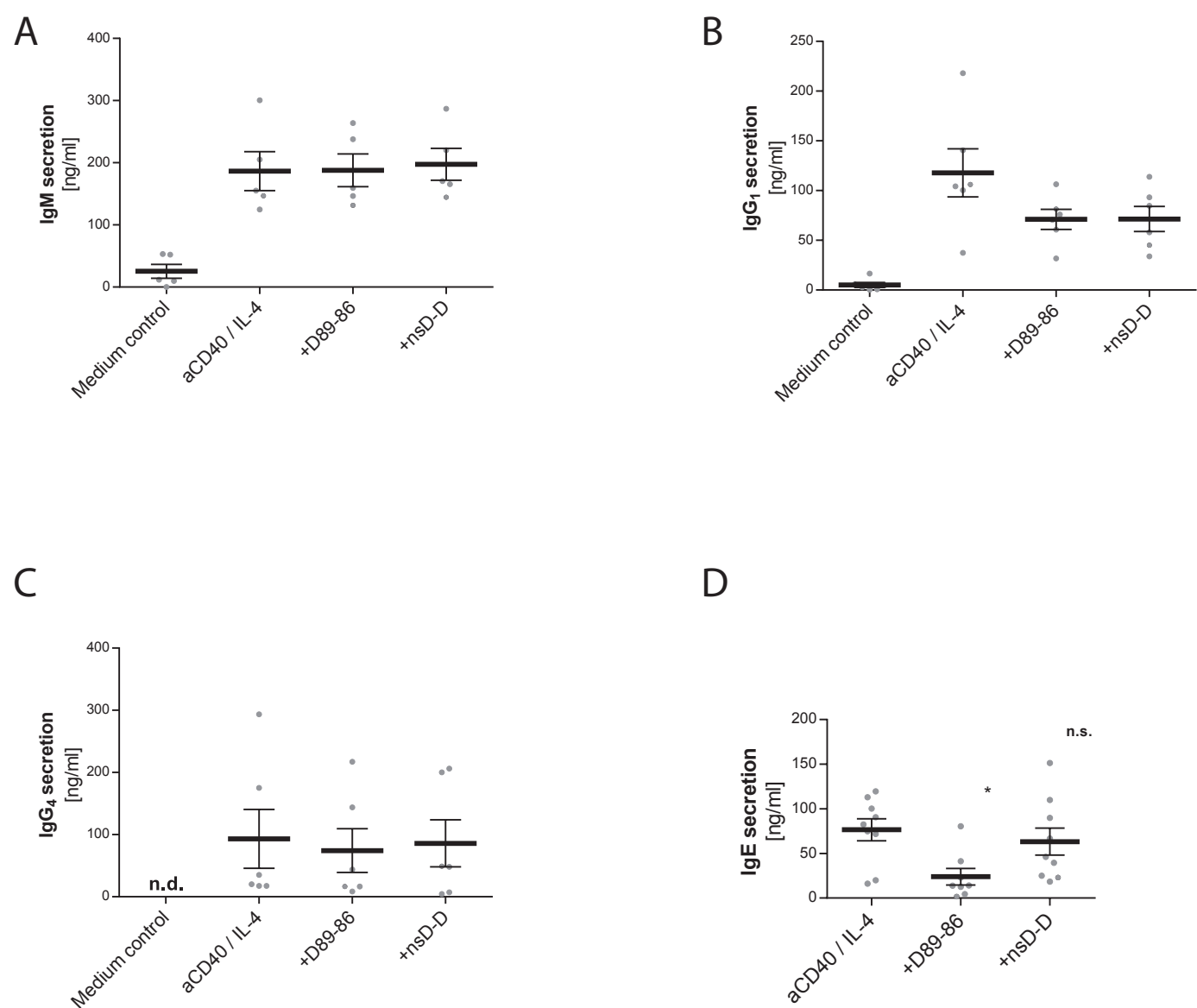

Supplement: Supplementary file 2 — Figure S1. Epitope specificities of anti‐CD23 DARPins. The chip was saturated with bivalent DARPin D86‐86, which has a low off‐rate dissociation constant. Figure S2. Protein analysis of anti‐CD23 DARPins. Anti‐CD23 DARPins (1.5 μg) were visualized on a 15% SDS‐PAGE stained with Coomassie. Figure S3. SPR sensorgrams for the assessment of binding kinetics of anti‐CD23 DARPins to CD23. Figure S4. Expression of CD20 and CD23 on freshly isolated B cells. Figure S5. Effects of anti‐CD23 DARPins on surface CD23 after 6 days of culture. Figure S6. Effects of anti‐CD23 DARPins on different immunoglobulin isotypes. [file IID3-3-339-s002.pdf]
